# Premature ventricular contraction–induced ventricular dysfunction in children without structural heart disease: a systematic review and meta-analysis

**DOI:** 10.1093/europace/euaf167

**Published:** 2025-08-11

**Authors:** Francesco Flore, Michele Lioncino, Marianna Cicenia, Daniele Garozzo, Cristina Raimondo, Corrado Di Mambro, Massimo Stefano Silvetti, Fabrizio Drago

**Affiliations:** Paediatric Cardiology and Cardiac Arrhythmias Complex Unit, Bambino Gesù Children’s Hospital, IRCCS, Piazza di Sant’Onofrio, 4, Rome 00165, Italy; Paediatric Cardiology and Cardiac Arrhythmias Complex Unit, Bambino Gesù Children’s Hospital, IRCCS, Piazza di Sant’Onofrio, 4, Rome 00165, Italy; Paediatric Cardiology and Cardiac Arrhythmias Complex Unit, Bambino Gesù Children’s Hospital, IRCCS, Piazza di Sant’Onofrio, 4, Rome 00165, Italy; Paediatric Cardiology and Cardiac Arrhythmias Complex Unit, Bambino Gesù Children’s Hospital, IRCCS, Piazza di Sant’Onofrio, 4, Rome 00165, Italy; Paediatric Cardiology and Cardiac Arrhythmias Complex Unit, Bambino Gesù Children’s Hospital, IRCCS, Piazza di Sant’Onofrio, 4, Rome 00165, Italy; Paediatric Cardiology and Cardiac Arrhythmias Complex Unit, Bambino Gesù Children’s Hospital, IRCCS, Piazza di Sant’Onofrio, 4, Rome 00165, Italy; Paediatric Cardiology and Cardiac Arrhythmias Complex Unit, Bambino Gesù Children’s Hospital, IRCCS, Piazza di Sant’Onofrio, 4, Rome 00165, Italy; Paediatric Cardiology and Cardiac Arrhythmias Complex Unit, Bambino Gesù Children’s Hospital, IRCCS, Piazza di Sant’Onofrio, 4, Rome 00165, Italy

**Keywords:** Premature ventricular contractions, Ventricular dysfunction, Paediatrics, Arrhythmias, Structurally normal heart

## Abstract

**Aims:**

Premature ventricular contractions (PVCs) in paediatric patients often present a benign course. However, a minority of patients may develop left ventricular (LV) dysfunction, and risk factors are still under debate. The aim of this systematic review and meta-analysis was to analyse the prevalence of PVC-induced cardiomyopathy (CMP) and understand the risk factors in paediatric patients with PVCs and structurally normal hearts.

**Methods and results:**

A systematic search strategy was performed to identify original reports published between 1 January 2000 and 31 August 2024. Studies including adult patients and patients with cardiomyopathies, congenital heart diseases, or channelopathies were excluded. Seventeen studies were included and comprised 1.701 patients, with a mean age of 11.4 years. The mean burden of PVCs across the included studies was 16% (12.2–19.7). Left ventricular systolic dysfunction occurred in 40 patients, and they showed older age at presentation. Premature ventricular contraction burden emerged as significant risk factor for PVC-induced CMP (mean burden among patients with and without LV dysfunction 32.5 and 15.47%, respectively). Shorter coupling intervals and longer QRS duration were predictors in a few studies. No major adverse cardiovascular events occurred. Left ventricular dysfunction recovered in all but one patient after spontaneous or pharmacologically induced PVC reduction. Class IC drugs showed greater efficacy than other drugs.

**Conclusion:**

Premature ventricular contraction–induced CMP is rare in children, and PVC burden is the key determinant of risk. The threshold burden associated with LV dysfunction is higher in paediatric patients than in adults. Most patients with PVC-induced CMP experience normalization of LV function during follow-up.

## Graphical Abstract

**Graphical Abstract:**
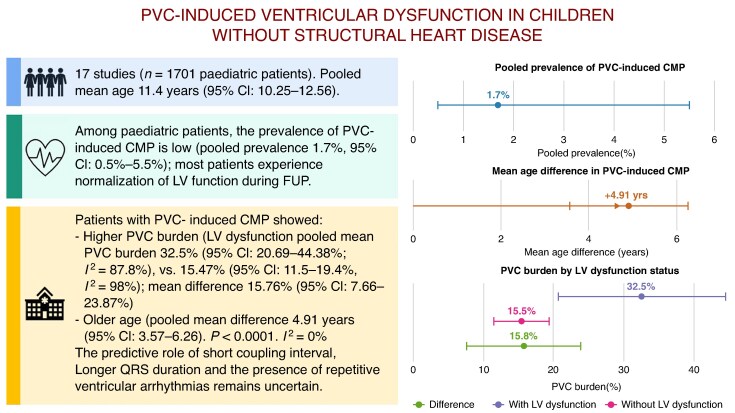

What’s new?

- In children, the prevalence of left ventricular (LV) dysfunction among patients with premature ventricular contraction (PVC) is low (pooled prevalence 1.7%), both when considering studies that included patients with a PVC burden greater than 10% and those that did not.
- The main predictor of LV dysfunction is a high PVC burden [mean burden in paediatric patients with LV dysfunction 32.5% with 95% confidence interval (CI) 20.69–44.38% vs. 15.47% with 95% CI 11.5–19.4% in patients without LV dysfunction].
- The occurrence of PVC-induced cardiomyopathy (CMP) is also associated with an older age and the male sex, while the predictive role of a short coupling interval, a longer QRS duration, and the presence of repetitive ventricular arrhythmias is not consistently demonstrated across the studies.
- Most patients with PVC-induced CMP experience normalization of LV function during follow-up, either with a spontaneous reduction of PVCs or due to pharmacological/ablation therapies.
- Class IC drugs show greater efficacy than other drugs in reducing PVC burden and, consequently, PVC-induced CMP.
- Transcatheter ablation was reported to be effective in every reported case, with only one probable significant adverse event.

## Introduction

Premature ventricular contractions (PVCs) are observed in paediatric patients with a reported incidence of 20–25%^1^ and often represent an incidental finding on electrocardiogram (ECG).

A substantial percentage of PVCs disappears over time, especially when first diagnosed in small children.^2^ A minority of patients may develop left ventricular (LV) systolic dysfunction.^3–5^ Risk factors of LV dysfunction in adults with PVCs and a structurally normal heart include high PVC burden, short coupling interval, prolonged QT interval, retrograde P-waves, duration of PVCs, and the presence of VTs.^5,6^ On the other hand, in children, risk factors for developing LV dysfunction are still poorly understood. Consequently, what is the most appropriate management of asymptomatic PVCs in children remains an unsolved issue.

The aim of this systematic review and meta-analysis was to analyse the prevalence of PVC-induced cardiomyopathy (CMP) and understand the risk factors in paediatric patients with PVCs and structurally normal hearts.

## Methods

### Literature sources and search strategy

This systematic review and meta-analysis was conducted in accordance with the Preferred Reporting Items for Systematic Reviews and Meta-Analysis (PRISMA) 2009 guidelines.^7^ The protocol for this systematic review and meta-analysis is registered in the international prospective register of systematic reviews (PROSPERO) (registration number: CRD42024611475). A systematic search strategy was performed in Medical literature analysis and retrieval system online (MEDLINE), Excerpta Medica database (EMBASE), Cochrane Register of Controlled Trials, and Web of Science databases, to identify original reports published between 1 January 2000, and 31 August 2024. The search string used to perform the systematic review was (*Premature ventricular contraction OR Premature ventricular beat OR Premature ventricular complex OR heart ventricle extrasystole) AND (child OR children*). The bibliographies of all the identified original studies and review articles were analysed to check for additional studies of interest. Two independent investigators (F.F. and M.L.) reviewed all citations retrieved from the electronic search to identify potentially relevant articles. Two authors independently reviewed the potential studies to determine their eligibility for inclusion in the screening phase. Disagreements were resolved by collegial discussion with a third co-author (F.D.).

### Inclusion and exclusion criteria

Retrospective and prospective cohort studies, cross-sectional studies, randomized controlled trials, and case series were included in the analysis. All studies that were included were peer-reviewed and only enrolled children without clinically apparent structural disease or primary electrical disease. We did not include in our analysis (i) studies enrolling patients ≥ 18 years or with cardiomyopathies, congenital heart diseases, or channelopathies; (ii) studies not reporting echocardiographic evaluation during clinical assessment or follow-up; (iii) papers whose full text was not available in the English language; and (iv) conference abstracts, letters, comments, editorials, case reports, systematic reviews, and meta-analysis.

### Data extraction and quality assessment

We extracted data on study design, inclusion and exclusion criteria, year of publication, participant characteristics (population selection, size, mean age, sex), mean PVC burden, prevalence of PVC-induced CMP, symptoms, PVC morphology, coupling rate, QRS duration, pharmacologic therapy, and follow-up data. Data extraction forms were completed by two authors (M.L and F.F) and verified for completeness and accuracy by a third author (F.D). The quality of the included studies was assessed through the Newcastle–Ottawa scale (NOS) for observational studies.^8^ Each eligible article was reviewed by two authors, who independently rated its quality according to the following items: representativeness of the study cohort, inclusion criteria of the examined population, assessment mode of candidate predictors associated with PVC-induced CMP, comparability of patients with CMP and controls, assessment of outcome (LV dysfunction), and duration of the follow-up. Non-randomized studies were considered high quality when NOS was ≥7, moderate quality when NOS was 5–6, and poor quality when it was <5. Disagreements about quality assessment were resolved by a third reviewer.

### Statistical analysis

Demographic data were extracted and pooled from the included studies. For studies reporting medians and interquartile ranges, we applied the Wan method^9^ or Hozo’s correction^10^ to convert these estimates into means ± standard deviations. The *I*² statistic was used to quantify the proportion of the total variance explained by between-study heterogeneity and was categorized as low (*I*² = 25–50%), moderate (*I*² = 50–75%), or high (*I*² ≥ 75%), in accordance with the classification criteria proposed by Higgins *et al*. Between-study variance was further quantified using τ². All meta-analyses were conducted using a random-effects model, except in cases involving only two studies.^11^

Prior to data pooling, influence analyses were performed to identify outliers, with Baujat and leave-one-out plots generated for visualization. A risk or protective factor was subjected to meta-analysis when data from at least two studies were available. All meta-analyses were conducted using univariate data, thereby providing unadjusted pooled estimates. For meta-analysis of proportions (i.e. prevalence), we used a generalized linear mixed-effects model with a random intercept logistic regression approach. Proportions were logit-transformed and pooled estimates were back-transformed to obtain summary proportions. Between-study variance was estimated using the maximum likelihood method. Continuity correction of 0.5 was applied to studies with zero events.

For continuous variables, we used a random-effects model, and effect sizes were pooled via the inverse variance method. Between-study variance (τ²) was estimated using the restricted maximum likelihood (REML) approach, and 95% confidence intervals (CI) were adjusted using the Hartung–Knapp method. To compare differences in continuous variables between two groups, we used inverse variance methods with REML estimator for τ², and CIs in the random-effects model were based on the standard normal distribution. For the meta-analysis of binary outcomes [odds ratios (ORs)], results were pooled using the inverse variance method under a random-effects model. Between-study variance (τ²) was estimated using the Paule–Mandel method, and the CI for τ² was obtained using the Q-profile method. The Hartung–Knapp adjustment was applied for CIs.

As an exploratory measure, the standardized mean difference (SMD) was also calculated to present an additional common effect size across studies. For statistically significant results, publication bias was assessed visually using funnel plots. When analyses included at least 10 studies, funnel plot asymmetry was formally evaluated using the Egger test or Peter’s regression, as appropriate. In cases where reporting bias was detected, the Duval and Tweedie trim-and-fill method was applied to estimate bias-corrected effect sizes.

For meta-analyses with significant heterogeneity, we conducted prespecified sensitivity analyses using the trim-and-fill method, excluding relevant outliers. Additional sensitivity analyses were performed for meta-analyses with a small number of studies by removing outliers. Random-effects meta-regressions were undertaken to examine whether relevant covariates were associated with pooled effect estimates or explained heterogeneity. Random-effects subgroup analyses were also conducted to assess the potential influence of study quality, methods for detecting LV systolic dysfunction [e.g. LV ejection fraction (EF) vs. shortening fraction (SF)], and study inclusion criteria (e.g. inclusion of patients with a PVC burden > 10% vs. inclusion irrespective of burden).

All analyses were conducted using R software (version 4.4.1; R Foundation for Statistical Computing, Vienna, Austria). Meta-analyses were performed using the meta package (version 8.1-0) and the metafor package (version 4.8-0).^12,13^ A result was considered statistically significant if *P* < 0.05.

## Results

### Summary and quality of the studies

**Figure 1:**
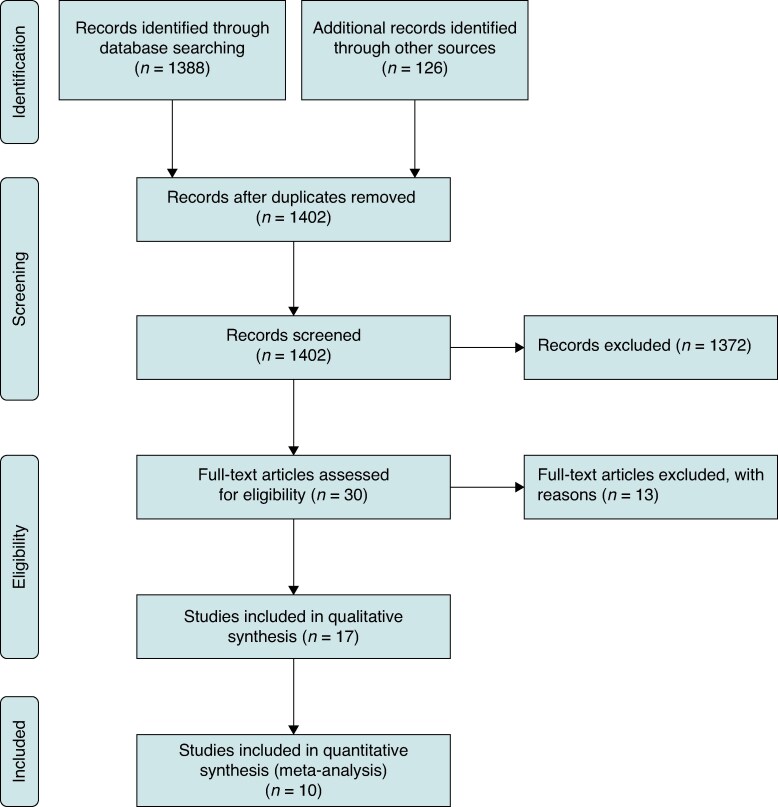
PRISMA flow diagram.

The literature search initially identified 1514 studies. After removing duplicates and manuscripts not meeting the inclusion criteria, 17 studies were included (*Table 1* and *Figure 1*).

**Table 1:** Key features of studies included in the meta-analysis

Study	Year	Journal	Sample size	Type of study	Aim of the study	Recruitment period	Inclusion	Exclusion	Definition of LV dysfunction	Follow-up
Abadir *et al*.	2016	*Heart Rhythm*	47	Single-centre cohort study	Assess the prevalence of LV systolic dysfunction in children with frequent PVCs	2008–2012	Children (age ≤ 18 years) with frequent PVCs (≥10%)	Significant structural cardiac anomalies	LV SF *z*-score < −2	4.0 ± 2.8 years
Bansal *et al*.	2020	*Pediatric Cardiology*	29	Data from an electronic medical record provided by Holter monitoring companies	The goal of this investigation is to determine whether children with PVCs may have myocardial dysfunction as measured by STE, despite normal standard echocardiographic measurements.	NA	Paediatric patients (age < 21 years) with ≥5% PVCs on 24 h Holter monitor	All patients with known history of cardiomyopathy, congenital heart disease, and systemic conditions affecting cardiac function such as lupus or hypertension or history of cardiopulmonary resuscitation	LV ejection fraction (LV EF) ≤ 55%	NA
Beaufort-Krol *et al*.	2008	*Europace*	59	Single-centre, retrospective cohort	To study the natural history of PVCs in childhood and whether there is a difference between PVCs originating from the right or the left ventricle	Since 1975	Children with frequent PVCs and anatomically normal heart	NA	NA	3.1 + 3.1 years
Bertels *et al*.	2024	*Heart Rhythm*	19	Open-label, prospective, randomized, multicentre, crossover trial	To further investigate the effect of beta-blockers compared with flecainide in reducing the PVC burden	September 2018 to December 2022	Age 1 year and <18 years, structurally normal heart, and PVC burden > 15% on two different 24 h Holter recordings, with or without idiopathic VT. Successively treated with metoprolol and flecainide	Structural cardiac defects, history of cardiac surgery, myocarditis, cardiomyopathies, inherited arrhythmia syndromes, verapamil-sensitive PVC/VT, and psychomotor mental retardation	NA	Weeks
Bertels *et al*.	2015	*Europace*	72	Single-centre cross-sectional study	To assess which determinants of asymptomatic PVCs/VTs are associated with development of LV dysfunction in children	NA	All children with frequent monomorphic PVCs (defined as.5% PVC burden on a Holter recording) with or without asymptomatic VTs	Patients with a structural heart disease, a history of cardiac surgery, myocarditis, cardiomyopathies, prolonged QT syndrome, or verapamil-sensitive PVCs/VTs	LV SF of <28%	3.8 (+4.1)
Doctor *et al*.	2010	*Pediatric Cardiology*	91	Single-centre retrospective review	The primary objective of our study was to evaluate if there is an association between exercise stress parameters and PVC burden detected on a 24 h Holter in children without structural heart disease.	From January 2000 to December 2018	Patients < 21 years of age with PVCs who had a 24 h Holter and exercise stress test	Patients with congenital heart disease, cardiomyopathy, known channelopathy, or incomplete medical records were excluded from the study. Patients who did not undergo exercise testing, Holter monitoring, or echocardiography within 1 week of each other were also excluded from study.	LV SF of <28%	NA
Guerrier *et al*.	2015	*Am J Cardiol*	123	Retrospective cohort study	The purpose of this study was to examine the relation between PVC burden on 24 h Holter monitoring and LV systolic function on echocardiography in paediatric patients with structurally normal hearts.	From January 1998 to June 2013	Patients aged 0 to 21 years who underwent 24 h ambulatory electrocardiographic (Holter) monitoring that showed 0.5% PVCs	Patients with known channelopathies, cardiomyopathies diagnosed before the initial Holter study, ventricular tachycardia, and/or congenital heart disease were excluded.	LV SF of <28%	13–14 months
Kakavand et al.	2009	*Pediatr Cardiol*	28	Retrospective cohort	This study aimed to delineate further the incidence of PVC-induced cardiomyopathy in a subset of patients with frequent PVCs, the morphology of the PVCs, and the outcome of treatment for children.	Between 2003 and 2007	Patients’ ages 1 day to 18 years with the diagnosis of PVCs. The inclusion criteria required up to two PVC morphologies and at least a 5% ectopy burden on a 24 h Holter monitor.	Concomitant congenital heart disease (except for a patent foramen ovale), a history of surgical repair for a congenital heart disease, cardiomyopathies, and ‘burnt-out’ myocarditis	An LV fractional shortening <30% was considered abnormal in the newborn period, with a 28% rate considered abnormal thereafter.	2.7 ± 2.3 years
Pietrzak *et al*.	2023	*Heart Rhythm*	49	Prospective case-control	This study aimed to determine whether monomorphic ventricular arrhythmia affects physical performance in adolescents with normal left ventricular function, using a cardiopulmonary exercise test (CPET) and evaluating the electrocardiographic (ECG) characteristics of patients with PVCs with regard to exercise capacity.	Between March 2021 and March 2022	Patients aged 8–17 years with frequent, monomorphic, idiopathic PVCs and normal left ventricular function	NA	Left ventricular ejection fraction < 50% with abnormal LVDd (normal *z*-score values −2 to +2)	NA
Porcedda *et al*.	2020	*Pediatric Cardiology*	103	Prospective cohort study	–	From 1996 to 2016	–	–	–	
Przybylski *et al*.	2024	*Europace*	198	Single-centre retrospective cohort study	We sought to characterize the natural history of PVCs in childhood including the prevalence of, and factors associated with, LV dysfunction.	From 2003 to 2018	Patients aged 1–21 years with frequent PVCs (>0.5% on 24 h Holter monitoring), a 12-lead ECG with ≥1 PVC, and an echocardiogram	Patients with congenital heart disease aside from minor septal defects and valvular abnormalities and those with known personal or family history of primary electrical or cardiomyopathic disease were excluded.	LV ejection fraction (EF) < 50%	3.6 years, IQR 2.2–6.5 years
Sharma *et al*.	2019	*Ann Noninvasive Electrocardiol*	134	Retrospective review of Holter studies	To validate the association of cardiac dysfunction and ventricular tachycardia with high PVC burden. To the association or progression of PVC burden and any predictive risk factors for the same (extent of PVC burden, presence with exercise, morphology, and association with stimulants or medications)	Between December 2015 and December 2017	Patients from 6 months to 18 years of age were included with structurally normal heart and PVCs.	CHD, vascular rings/slings, history of CBG or ECMO, CMPs. Family history of CMP or channelopathy, coronary abnormalities, previous chemotherapy. Patients with SVT, heart block, or sinus node dysfunction were also excluded.	Ventricular shortening fraction < 28%, left ventricular ejection fraction < 54%, left ventricular end-diastolic z-scores > 2, or diminished function on qualitative interpretation	
Sun *et al*.	2003	*International Journal of Cardiovascular Imaging*	40	Single-centre cross-sectional study	To assess the influence of PVCs on echocardiographic left ventricular function and cardiac index	NA	Asymptomatic children with isolated monomorphic PVCs without structural heart disease	Children with structural heart disease	NA	NA
Tosyalı *et al*.	2024	*J Pediatr Res*	60	Single-centre retrospective study (cohort?)	This study aimed to determine the clinical progression of PVCs in children without any underlying cardiac structural abnormalities and assess the necessity and effectiveness of medical treatment.	NA	Children younger than 18 years of age who were diagnosed with PVCs	This study did not include paediatric patients with structural heart disease, a history of heart surgery, malignant ventricular arrhythmia, a history of stimulant drug use, any underlying chronic disease, or a family history of major arrhythmia. Moreover, newborns were also excluded from this study.	NA	18 months (min 3–max 120)
Uysal *et al*.	2023	*Turkish Archives of Pediatrics*	226	Single-centre retrospective	To assess the clinical course of PVC in children without cardiac structural abnormalities and to evaluate the effectiveness of medical treatment	Between December 2010 and May 2022.	Patients aged 6 months to 18 years who underwent 24 h ambulatory electrocardiographic (Holter) monitoring	CHD, history of cardiopulmonary bypass, CMPs, channelopathies, coronary anomalies, and previous chemotherapy. Patients with a history of sinus node dysfunction, supraventricular tachycardia, heart block, or family history for CMP or channelopathy were also excluded from the study.	LVEDD *z*-score > 2 or reduced shortening fraction	8.7 ± 3.7 years
West *et al*.	2015	*The Journal of Pediatrics*	219	Single-centre retrospective	To describe the presentation and clinical course of patients with VE without known heart disease seen at a single institution	Between 1965 and 2015	Patients aged <18 years with documented PVC during ECG or Holter monitoring	Haemodynamically significant CHD; cardiac tumour; channelopathies; chest wall trauma; systemic (e.g. HIV, cystic fibrosis). Patients with CMPs and PVCs were excluded if the initial diagnosis of cardiomyopathy preceded the discovery of PVCs.	NA	3.1 years (IQR 0–21)
Zebulon *et al*.	2014	*Cardiology in the Young*	36	Single-centre retrospective	To examine the prevalence of PVC-induced tachycardia, natural history of PVCs, and progression to ventricular tachycardia	Between 1990 and 2013	Children and adolescents aged 6 months to 18 years with PVC burden > 20% during Holter monitoring	Patients with structural heart disease, family history of cardiomyopathy of major systemic disease were excluded. Patients with VT lasting >1% of total daily rhythm were excluded.	Left ventricular fractional shortening ⩽ 28%, left ventricular ejection fraction ≤ 54%, left ventricular end-diastolic dimension *z*-score ⩾ 2.2, or globally diminished function on qualitative interpretation	NA

There were nine single-centre retrospective cohort studies, six cross-sectional studies (five retrospective and one prospective), one open-label, multicentre randomized crossover trial, and one prospective cohort study. According to the NOS, six studies presented low risk of bias (NOS ≥ 7), eight studies moderate risk of bias (NOS 5–6), and three studies were of poor quality.

The PVC-induced CMP was defined as a SF ≤28% or a LV shortening *z*-score ≤ −2 in eight studies.^1,3–5,14–17^ In two studies,^4,17^ CMP was assessed using both SF and EF. Conversely, in three studies,^6,18,19^ PVC-induced CMP was defined as an EF ≤ 54% or ≤50%, respectively.

Four studies^4,16,19,20^ required a mean PVC burden ≥ 10% for inclusion. Among the included studies, the study by Beaufort-Krol *et al*.^2^ was based on the follow-up of a longitudinal series and the evolution of PVC morphology over time. For this reason, it was excluded from the quantitative analyses. Similarly, the studies by Sun *et al*.^21^ and Porcedda *et al*.^22^ quantified premature ventricular complexes using either the number of PVCs per minute or the 24 h count. As these measures were not comparable to those in the other studies, they were also excluded from the quantitative analysis.

### Characteristics of the study population

The characteristics of eligible studies are summarized in *Table 1*. Between 2000 and 2024, 17 studies were included, focusing on the association between PVCs and LV systolic dysfunction in paediatric patients. The study population comprised 1,701 patients (*Table 2*). Pooled mean age was 11.4 years (95% CI: 10.25–12.56). The proportion of male patients ranged from 50 to 86%. Follow-up data were available in 10 studies, ranging from 2 weeks to 8.7 ± 3.7 years.

**Table 2:** Baseline characteristics of patients in the included studies

Study	Group	Sample size	Age (mean)	Male (%)	Symptoms	FH of SCD	ADHD on therapy
Abadir *et al*.	All (mixed group)	47	8.2 ± 6.5	53	3	2	10
Bansal *et al*.	Normal cardiac function	29	11.7 ± 5.8	59	0	NA	NA
Beaufort-Krol *et al*.	Normal cardiac function	59	7.1 + 4.3	59	8	0	NA
Bertels *et al*. (ECTOPIC trial)	Normal cardiac function	19	13.9 (7.9–18.1)	58	9	NA	NA
Bertels *et al*.	Normal cardiac function	66	8.2 (+5.8)	58	22	NA	NA
Bertels *et al*.	LV dysfunction	6	10.3 (+6.8)	50	2	NA	NA
Bertels *et al*.	All						
Doctor *et al*.	Normal cardiac function	91	14.5 ± 3.2	61	52	NA	NA
Doctor *et al*.	Normal cardiac function (subgroup PVC > 10%)	48	14.3 ± 3.1	60	26	NA	NA
Guerrier *et al*.	Normal cardiac function	123	12 (6–14)	63	0	NA	NA
Kakavand *et al*.	Normal cardiac function	24	NA	61	NA	NA	NA
Kakavand *et al*.	LV dysfunction	4	9	50%	NA	NA	NA
Kakavand et al.	All	28	13.3 ± 5.9 years for males, 13 ± 5.2 years for females	61%	11	NA	NA
Pietrzak *et al*.	Normal cardiac function	49	13 ± 3	51%	0	0	NA
Porcedda *et al*.		103		66%			
Przybylski *et al*.	Normal cardiac function	191	12.1 (7.8–15.6)	57%	NA	NA	NA
Przybylski *et al*.	LV dysfunction	7	17.6 (16.2–17.6)	86%	NA	NA	NA
Przybylski *et al*.	All	198	12.3 (IQR) 7.8–15.8	58%	NA	NA	NA
Sharma *et al*.	All	134	10.5 years (IQR 6.1–14.8)	54%	NA (4 syncope)	NA	6
Sun *et al*.	All	40	6 range (3–12)	55	0	NA	NA
Tosyalı *et al*.	Normal cardiac function	60	10.75 (min 6 max 15)	55%	32	NA	NA
Uysal *et al*.	All	226	11.9 ± 3.9	66	41 (18%)	NA	NA
Uysal *et al*.	LV dysfunction	4	13.5 (±3.1)	NA	NA	NA	NA
West *et al*.	All	219	11.3 (range 0–26)	59%	137	NA	2
West *et al*.	LV dysfunction	4	15 IQR (11.75–16)	75	NA	NA	NA
Zebulon *et al*.	All	36	11 ± 4	NA	29	NA	2

The pooled mean burden of PVCs across the included studies was 16% (95% CI: 12.2–19.7), with values ranging from 8.2 to 28.65%. The weighted prevalence of symptomatic patients was 15% (95% CI: 4.2–41%). Four studies^4,17,18,23^ specifically included paediatric patients treated for attention-deficit/hyperactivity disorder (ADHD).

### Prevalence of premature ventricular contraction–induced cardiomyopathy

**Figure 2:**
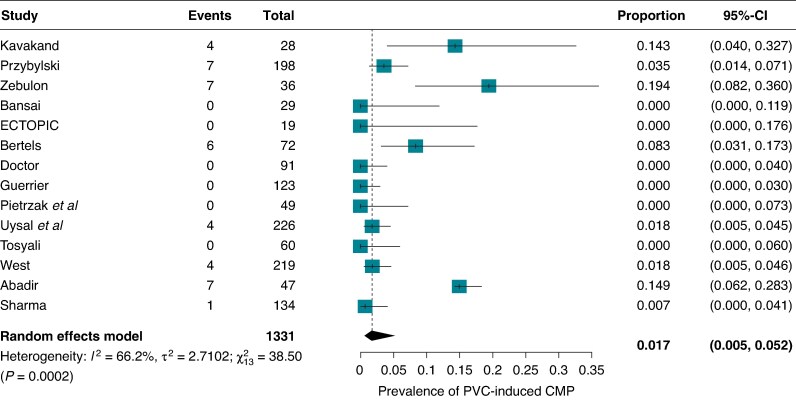
Forest plot of the mean prevalence of PVC-induced CMP among the included studies.

**Figure 3:**
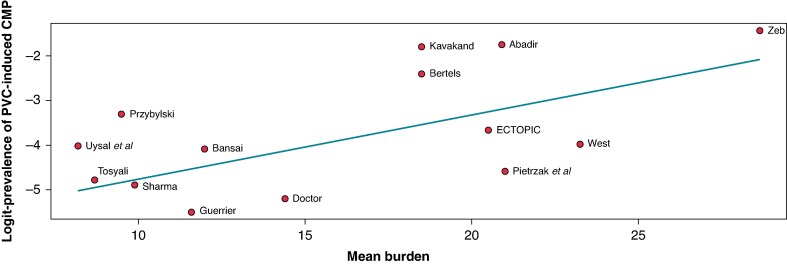
Meta-regression analysis showing the impact of mean PVC burden on the prevalence of PVC-induced CMP in the included studies.

Overall, 40 patients experienced LV systolic dysfunction, with a pooled prevalence of 1.7% (95% CI: 0.5–5.5%) (*Figure 2*). There was moderate heterogeneity (*I*² = 66.2%). A dose-response meta-regression analysis found that studies with higher PVC burden reported a higher prevalence of PVC-induced CMP (*Figure 3*). This model explained a significant portion of the variability, though substantial heterogeneity remained (76.59%). Specifically, for every 1-unit increase in mean burden, the logit-transformed prevalence increased by 0.14 (95% CI: 0.02–0.26). The estimated prevalence remained consistent across different modifiers in both univariate meta-regression and subgroup analyses (see *Table 3* and Supplementary material online, *Figures S1*  *and S2*).

**Table 3:** Meta-regression analysis examining impact of covariates on the prevalence of PVC-induced CMP

	Estimate	95% CI	*P*-value
Age	−0.4222	(−0.9559, 0.1114)	0.1210
Year of publication	−0.1422	(−0.3319, 0.0474)	0.1416
Gender (male)	−3.9274	(−28.0736, 20.2187)	0.7499
Symptoms	2.3175	(−1.8942, 6.5292)	0.2808
NSVT	−0.0096	(−0.0942, 0.0751)	0.8246

The funnel plot suggested potential publication bias (Peter’s test, *P* = 0.004; Supplementary material online, *Figure S3*). To address this, we calculated trim-and-fill adjusted estimates (see Supplementary material online, *Figure S4*). In addition, we performed a sensitivity analysis with the exclusion of three studies^3,4,16^ and the results were consistent.

### Association between age and prevalence of left ventricular systolic dysfunction

**Figure 4:**
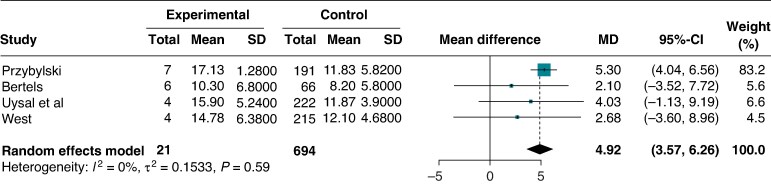
Forest plot of the mean age difference between patients with and without PVC-induced CMP.

The association between age and LV systolic dysfunction was reported in four studies (*n* = 715). Patients with PVC-induced CMP showed older age at presentation [mean difference 4.91 years (95% CI: 3.57–6.26), *P* < 0.0001, *I*^2^ = 0%] (*Figure 4*).

### Role of burden in premature ventricular contraction–induced cardiomyopathy

A comparison of mean PVC burden between patients with and without PVC-induced CMP was reported in 6 out of 17 studies (779 patients). Among these are the following:

- Patients with LV dysfunction (*n* = 34) had a pooled mean PVC burden of 32.5% (95% CI: 20.69–44.38%; *I*² = 87.8%).
- Patients without LV dysfunction (*n* = 1297) had a pooled mean PVC burden of 15.47% (95% CI: 11.5–19.4%; *I*² = 98%).

**Figure 5:**
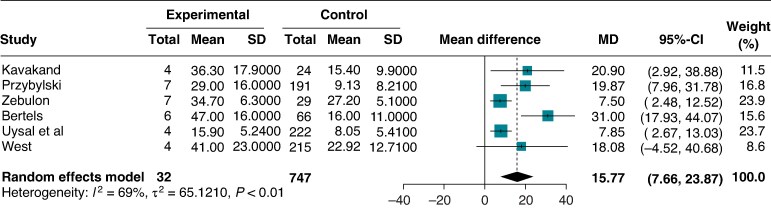
Forest plot of the mean burden difference according to the presence of PVC-induced CMP.

The pooled mean difference among the two groups was 15.76% (95% CI: 7.66–23.87%) (*Figure 5*). Heterogeneity was moderate (*I*^2^ = 68%). The SMD, calculated to provide an additional common effect size across pooled estimates, demonstrated a significant impact of burden on the risk of PVC-induced CMP [SMD 1.86 (95%CI: 1.4–2.3), *P* < 0.001, *I*^2^ = 26%]. The difference in mean burden was not influenced by age at inclusion, year of publication, or symptom prevalence (*Table 4*). However, meta-regression showed a significant study-level effect of sex: in studies with a higher proportion of male participants, the difference in PVC burden between patients with PVC-induced CMP and those with normal LV function was lower (−18.96% per 10% increase in male proportion; *p* = 0.0007; residual heterogeneity 0%). The result remained consistent in subgroup analysis (see Supplementary material online, *Figure S5*). Moreover, in a sensitivity analysis, after removing an outlier study (Bertels *et al*.), the adjusted mean burden difference was 11% (95%CI 5.63–13.49, *I*^2^ = 33%).

**Table 4:** Meta-regression analysis examining impact of covariates in the mean burden difference among patients with and without PVC-induced CMP

	Estimate (95% CI)	*P*-value
Gender (male)	−1.89 (−2.9; −0.8)	0.0007
Age	−2.86 (−7.96; 2.22)	0.27
Year of publication	−0.39 (−2.08; 1.3)	0.65
Symptoms	−2.9 (−15; 0.34)	0.64

### Premature ventricular contraction morphology

PVC morphology was assessed in five studies for LBB-PVCs (*n* = 560) and four studies for RBB-PVC (*n* = 362), respectively. Neither LBB morphology [OR: 1.1 (95% CI: 0.71–1.70), *P* = 0.56, *I*²=23%] nor RBB morphology [OR: 2.78 (95% CI: 0.17–43.49), *P* = 0.32, *I*²=54%] was significantly associated with a higher risk of PVC-induced CMP (see Supplementary material online, *Figure S6*). However, as our meta-analysis was underpowered to assess the impact of morphology (estimated power: 54 and 45%, respectively), the evidence is inconclusive, and our findings should be considered hypothesis-generating.

### QRS duration and coupling interval

Two studies^5,6^ provided data regarding QRS duration and the occurrence of PVC-induced CMP (*n* = 270). Patients with LV dysfunction showed higher mean QRS duration compared with their counterparts [mean difference 23.6 ms (95% CI 16.2–31), *P* < 0.0001], and no significant heterogeneity was detected (Cochran’s *P* = 0.15) (see Supplementary material online, *Figure S7*).

The impact of PVC coupling rate was assessed in two studies^5,6^; nevertheless, due to significant heterogeneity, meta-analysis could not be performed. In the study by Abadir *et al*.,^16^ coupling interval was found to be the best predictor of LV dysfunction and authors reported that a coupling interval ≤ 365 ms granted the greatest sensitivity/specificity for it. In the study by Sun *et al*.,^21^ children with frequent PVC (>14 400 per day), a shorter coupling interval ratio (<0.6) and a longer QT (>400 ms) had a lower LV EF (average of EF in sinus rhythm and during the PVC beat). On the other hand, according to Przybylski *et al*., PVC coupling rate was not significantly associated to the risk of LV dysfunction [OR: 0.9 (0.7–1.1) per 25 ms increase, *P* = 0.42].^6^ Similarly, in the study by Bertels *et al*., PVC coupling interval was not significantly lower among patients with SF < 28% compared with those with normal SF (mean 0.63 ± 0.14 vs 0.65 ± 0.13, *P* = 0.76).^5^ The absence of a significant association between coupling rate and LV dysfunction was also reported by Sharma *et al*. (OR 0.6, CI 95% 0.3–1.2).^17^

### Outcomes of premature ventricular contractions and premature ventricular contraction–induced cardiomyopathy

The natural history and outcomes of PVCs in paediatric patients with structurally normal hearts were assessed in 12 studies.^1–6,14,15,17,22–24^ Even though the rate of reduction/persistence of PVCs was variable among studies, no major adverse cardiovascular events were reported and PVC-induced CMP resolved in all but one patient. A meta-analysis on predictors of spontaneous reduction of PVCs and the consequent PVC-induced CMP was impossible to perform due to the heterogeneity and lack of specific data on this topic. However, two studies evaluated predictors of PVC burden reduction in both patients with and without LV dysfunction.^2,6^ Przybylski *et al*. showed that older age at diagnosis and presence of couplets were associated with a lack of significant PVC burden reduction, while in the study by Beaufort-Krol *et al*., PVCs originating from the right ventricle were more likely to persist with growth as compared to PVCs from the left ventricle.

Regarding the outcomes of PVC-induced CMP, they were assessed in five studies.^3,5,15,16,23^ Abadir *et al*. reported that during follow-up, PVC burden decreased along with a slight improvement of LV function (19.1% of patients were treated with antiarrhythmics at some point—mainly beta-blockers—and 6.3% underwent transcatheter ablation).^16^ In the study by Bertels *et al*., in 5/6 patients with LV dysfunction, cardiac function recovered at follow-up after appropriate treatment of PVCs, with Classes IC and II antiarrhythmic drugs in two and radiofrequency (RF) ablation in three. Regarding the patients who showed persistent LV dysfunction despite PVC suppression, authors speculate that it may be related to extensive scar created during four ablation procedures.^5^ Kakavand *et al*. reported that, among the four patients with LV dysfunction, in two, PVCs spontaneously reduced from 40 to 0% and from 27 to 2.8% (after ineffective medical therapies); in the other two, PVC burden was controlled with antiarrhythmic drugs. In all four patients, LV function normalized subsequently to decreases in PVC burden.^3^ In the study by Uysal *et al*.,^15^ LV dysfunction recovered in three out of four patients (two after RF ablation). In the study by West *et al*. at follow-up, LV function normalized in all patients with resolution of PVCs, while it developed in another patient whose burden had risen to 97% during follow-up.^23^

### The role of antiarrhythmic drugs

The role of antiarrhythmic drugs to reduce the PVC burden in paediatric patients was evaluated in six studies.^3,15,20,22–24^ Due to heterogeneity, meta-analysis could not be performed; however, overall, Class IC drugs were more effective in reducing PVC burden.

The ECTOPIC trial by Bertels *et al*. (a randomized open-label crossover trial) tested the efficacy of flecainide and metoprolol on PVC burden in patients with PVC burden > 15% on two different 24 h Holter recordings. The estimated mean reduction in PVC burden after 5 days of therapy was 10.6% (95% CI, 5.8–15.3) for flecainide and 2.4% (95% CI, 22.7–7.5) for metoprolol. After adjustment of the dose, 9/18 patients on flecainide obtained a PVC burden < 5%, while only 1/17 on metoprolol did the same. Beta-blockers were not effective in 3 out of 4 patients, while flecainide diminished the PVC burden to <1% in 1/1 patient.^3^ Porcedda *et al*. administered pharmacologic treatment in 7 (6.7%) patients (those with high-burden PVCs and/or clinical symptoms). Five received beta-blockers, one propafenone and another flecainide; all of them showed reduction of PVC burden and clinical symptoms.^22^ In the study by Tosyalı *et al*.,^24^ 40/60 patients were treated with antiarrhythmic and propafenone provided a more successful clinical response than beta-blockers. However, authors reported that among patients who did not receive any medical treatment, there was a higher percentage of a complete recovery. Thus, they concluded that antiarrhythmic treatment did not affect the course of the disease. In the study by Uysal *et al*., 31.8% of patients received medical therapy (most often beta-blockers, followed by calcium channel blockers and propafenone) and no significant difference was seen between treated and untreated group in terms of PVC burden reduction [from 20.2% (9.2–24.2) to 12.3% (5.3–15.2) in the treated group, from 10.9% (5.3–19.2) to 6.2% (1.3–11.2) in the untreated group (*P* = 0.46)].^15^

In the study by West *et al*., 31 children underwent medical therapy. Twenty-four patients were prescribed a beta-blocker; of these, 12 (50%) showed a decrease in PVC and six patients (25%) had side effects requiring cessation of drug treatment. A total of 16 patients were prescribed a calcium channel blocker; of these, seven (44%) showed a decrease in PVC and two patients (12.5%) had side effects requiring cessation of drug treatment.^23^

## Discussion

In our meta-analysis, the overall prevalence of PVC-induced CMP was found to be below 2%. Importantly, it must be acknowledged that there is lack of concordance among physicians on the definition of arrhythmia-induced CMP (and therefore also of PVC-induced CMP), as also highlighted by a recent EHRA survey.^25^ However, our finding was consistent both when using the EF and SF as the primary metric for detecting ventricular dysfunction. Moreover, it did not vary across subgroups, including subgroups differing for age at inclusion, sex, complexity of underlying arrhythmias, and selection criteria. Notably, in the subgroup analysis, no significant difference was observed between studies that exclusively included patients with a PVC burden greater than 10% and those that did not. This suggests that, while a linear association between PVC burden and the prevalence of CMP is evident, defining a clear threshold remains challenging. Additionally, it is important to highlight that the true prevalence of PVC-induced CMP may be even lower than reported. This is partly due to a general tendency in literature to overrepresent populations with higher PVC burdens and a greater number of clinical events, as evidenced by the presence of reporting bias. Also, in the studies analysed, no major adverse cardiovascular events occurred, and PVC-induced CMP resolved in all but one patient; this is in line with a recent study including adult patients with frequents PVCs and no evidence of structural heart disease, followed for around 5 years.^26^

Regarding predictors of PVC-induced CMP, we found that PVC burden seems to be the main determinant in LV systolic dysfunction. Notably, in adult patients, a PVC burden ≥ 10–20% is generally considered a significant risk factor.^27^ Despite that, we observed that the mean PVC burden was significantly higher in children with LV dysfunction (32.5%, 95% CI: 20.69–44.38%) compared to those with normal EF (15.47%, 95% CI: 11.5–19.4%). These findings carry important clinical implications, as they suggest that a PVC burden below 20% rarely leads to LV dysfunction in paediatric patients. Several mechanisms may explain this difference: in general, children may tolerate significantly higher heart rate ranges, and the absence of secondary causes of ventricular dysfunction such as myocardial ischaemia or fibrotic remodelling allows for an effective compensatory response to metabolically demanding conditions.^28–31^ This also raises important implications for therapeutic management, as it prompts a re-evaluation of the risk-benefit balance of antiarrhythmic therapy in children with PVCs.^32^

In our systematic review, we reported that age and sex seem to play a role in modulating CMP risk. Age was evaluated in four studies, and children with PVC-induced CMP were significantly older than those with preserved ventricular function. This finding may be explained by a cumulative effect of PVC burden over time. However, the paediatric population is highly heterogeneous, and age appears to be a critical modulator of the arrhythmic phenotype. In newborns with structurally normal hearts, PVCs often reflect immaturity of the cardiac conduction system and autonomic regulation, typically resolving spontaneously.^32^ In contrast, PVCs in post-pubertal adolescents are less likely to resolve and are more frequently associated with adverse outcomes, including LV dysfunction, as reported by Przybylski *et al*.^6^ Indeed, hormonal changes during puberty could play a role in modulating arrhythmic susceptibility. In this regard, we found that the impact of burden on the risk of PVC-induced CMP was modulated by sex. In particular, for each 10% increase in the proportion of males, the mean burden difference between patients with CMP and normal ventricular function decreased by 18%. Moreover, it may also be linked to differences in symptom perception, with women being more sensitive to PVCs and therefore seeking medical attention more easily.^33^ This finding is in line with previous studies in the adult population, reporting a significant association between male sex and the risk of developing of CMP.^34–36^

This review seems also to highlight the role of QRS duration and coupling interval in paediatric patients. Patients with LV dysfunction exhibited a longer PVC QRS duration. This is not surprising, since it is well known from adult studies that a dyssynchronous ventricular activation may result in changes in cardiac metabolism, perfusion, haemodynamics, and mechanical function.^37,38^ This can be also seen in patients with right ventricular pacing or in those with left bundle branch block.^39,40^ Coupling interval did not emerge as a consistent predictor of LV dysfunction, with highly heterogeneous results across studies. This variability is likely due to differences in how coupling interval was assessed and reported. While Abadir *et al*. demonstrated that a coupling interval cut-off of <365 ms had high sensitivity and specificity for predicting PVC-induced CMP, most other studies used coupling rate or other cut offs. The lack of standardized methods for evaluating coupling interval limits our ability to assess its true predictive value in this meta-analysis. Nevertheless, on a pathophysiological point of view, the role of coupling interval is controversial: in canine models, PVCs with a shorter coupling interval are associated with a lower cardiac output but less pronounced ventricular dyssynchrony.^41^

Regarding PVC morphology, neither LBBB nor RBBB-PVCs are associated with the risk of PVC-induced CMP, despite our study is underpowered to lead to this conclusion.

Another important finding is that, in our meta-analysis, we demonstrated that PVC-induced CMP has high likelihood of LV recovery during follow-up. Left ventricular function spontaneously recovered in several cases, while in others, improvement was associated with antiarrhythmic therapy or catheter ablation of PVCs. In this regard, based on available data on paediatric patients, it is impossible to pinpoint the predictors of spontaneous reduction of PVC burden with time, especially in patients with PVC-induced CMP. When considering both patients with and without LV dysfunction, older age at diagnosis seems to be the only reliable predictor of PVC persistence and consequent PVC-induced CMP, as suggested also by adults’ literature^6,42,43^; however, specific research on this topic is needed and, nowadays, it is reasonable to treat every patient with PVC-induced CMP.

As previously mentioned, and according to international guidelines, LV dysfunction associated with PVCs represents an indication for treatment.^27^ In this regard, Class IC drugs,^44^ and especially flecainide, show greater efficacy than other drugs. Antiarrhythmic drugs were used also in patients with normal LV function. However, they do not change the natural history of PVCs.^24^ Therefore, the role of preventive pharmacologic intervention remains to be further clarified. Conversely, ablative therapy has been reported to be truly effective when the indication is appropriate, but adverse events may occur.

### Limitations

Several limitations must be acknowledged.

First, the included studies exhibited significant heterogeneity in multiple aspects, including study design, population characteristics, and PVC burden as inclusion criteria (high burden vs. any burden). Similarly, the definition of PVC-induced CMP varied across studies, with some using LV SF ≤ 28%, others employing LV EF ≤ 50–54%, and some incorporating both criteria. To address these inconsistencies, we conducted subgroup analyses based on study quality, inclusion criteria, and diagnostic methods.

Another limitation regards study quality and risk of bias. The overall quality of the studies included was variable, with only six studies classified as having a low risk of bias (NOS score ≥ 7), eight studies at moderate risk (NOS 5–6), and three studies at high risk. The reliance on retrospective study designs (9/17 studies) introduces an inherent risk of selection bias, as patient inclusion may have been influenced by clinical suspicion rather than systematic screening. Additionally, five studies were single-centre retrospective cohorts, which may not be fully representative of the broader paediatric population.

Given the potential for publication bias (suggested by the funnel plot and Peter’s test), it is possible that the estimated association between PVC burden and CMP was inflated in this study. Even though we applied trim-and-fill corrections to adjust for publication bias, the robustness of our findings could be strengthened by future prospective, multicentre studies with predefined inclusion criteria.

Another potential study limitation pertains to the use of Wan *et al*.’s method for estimating the mean and standard deviation from the median and interquartile range. However, while this approach may introduce some approximation, the method demonstrates good performance for both normal and skewed data.

Lastly, although our meta-regression analysis identified a significant association between older age and increased risk of PVC-induced CMP, our ability to perform a detailed age-stratified analysis was limited by data availability. Only four studies explicitly examined the relationship between age and CMP, and none provided age-stratified results.

## Conclusion

This systematic review and meta-analysis shows that PVC-induced CMP is rare in children and that PVC burden is the key determinant of risk. The threshold burden associated with LV dysfunction is higher in paediatric patients (>30%) than in adults (10–20%), indicating a greater compensatory capacity in younger hearts. Older age and male sex may contribute to a higher susceptibility to PVC-induced LV dysfunction, but further studies are needed to clarify these associations. The role of QRS duration and coupling interval remains uncertain, though they may signal a higher risk of LV dysfunction.

When PVC-induced CMP occurs, treatment is needed. In these cases, Class IC drugs show greater efficacy than other drugs, and catheter ablation is an effective option, with only one adverse event reported in this series.

Regrettably, the overall quality of paediatric literature on PVC-induced CMP is poor, with only one randomized trial, and the studies exhibit significant heterogeneity in multiple aspects, including population characteristics. Therefore, future prospective, multicentre studies with predefined inclusion criteria and age-specific subgroups are warranted to better clarify the risk of developing PVC-induced CMP and its optimal management.

## Supplementary Material

euaf167_Supplementary_Data

## Data Availability

The data underlying this article will be shared on reasonable request to the corresponding author.
