# Supplementary material for: Premature ventricular contraction–induced ventricular dysfunction in children without structural heart disease: a systematic review and meta-analysis
Source: Europace. 2025 Aug 11;27(8):euaf167. doi: 10.1093/europace/euaf167 (PMC12395427; doi:10.1093/europace/euaf167)

**TABLES AND FIGURES – Supplementary**

***Supplemental Figures 1-2:*** *Subgroup analysis of the mean prevalence of PVC-induced CMP*

**
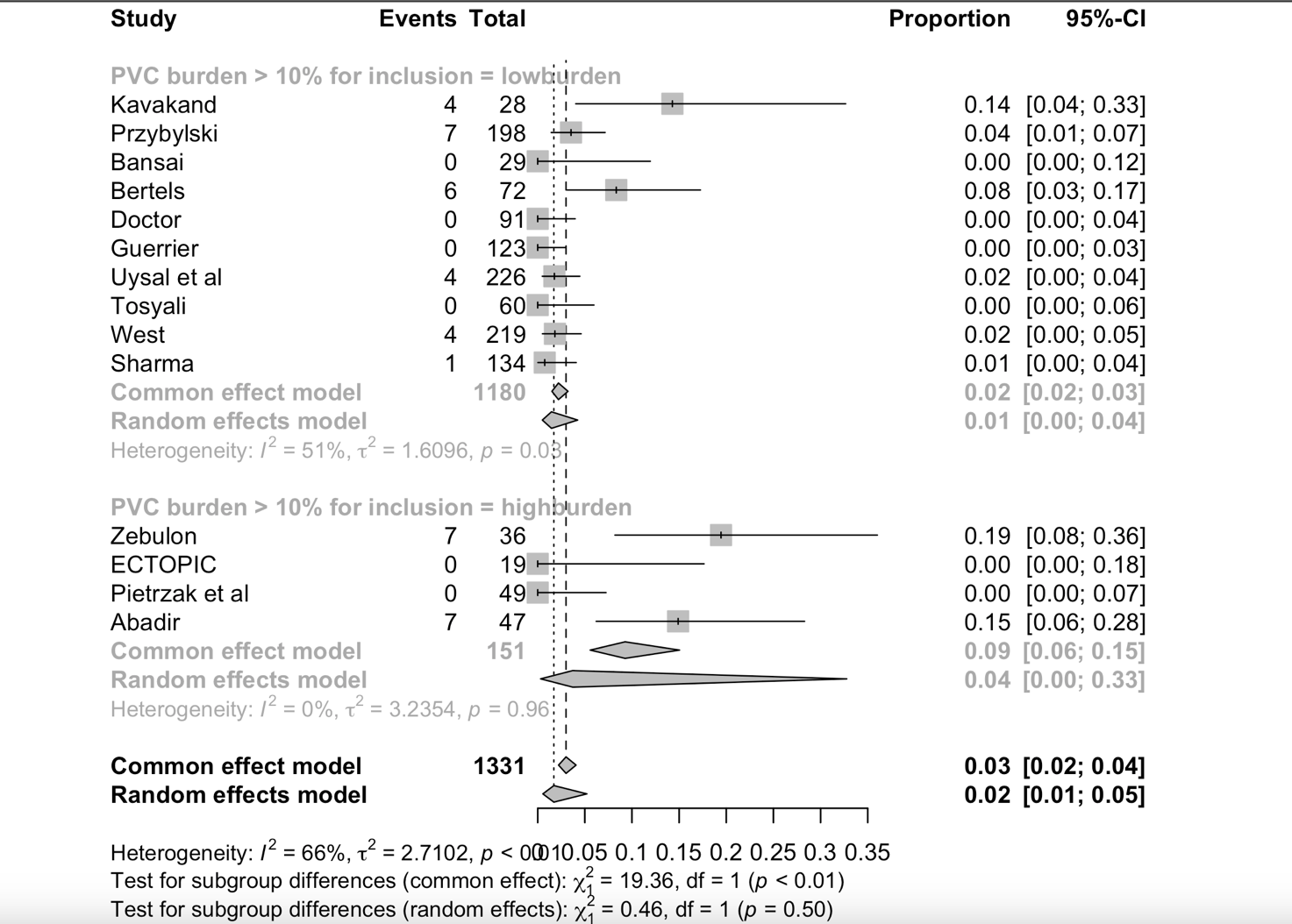
**


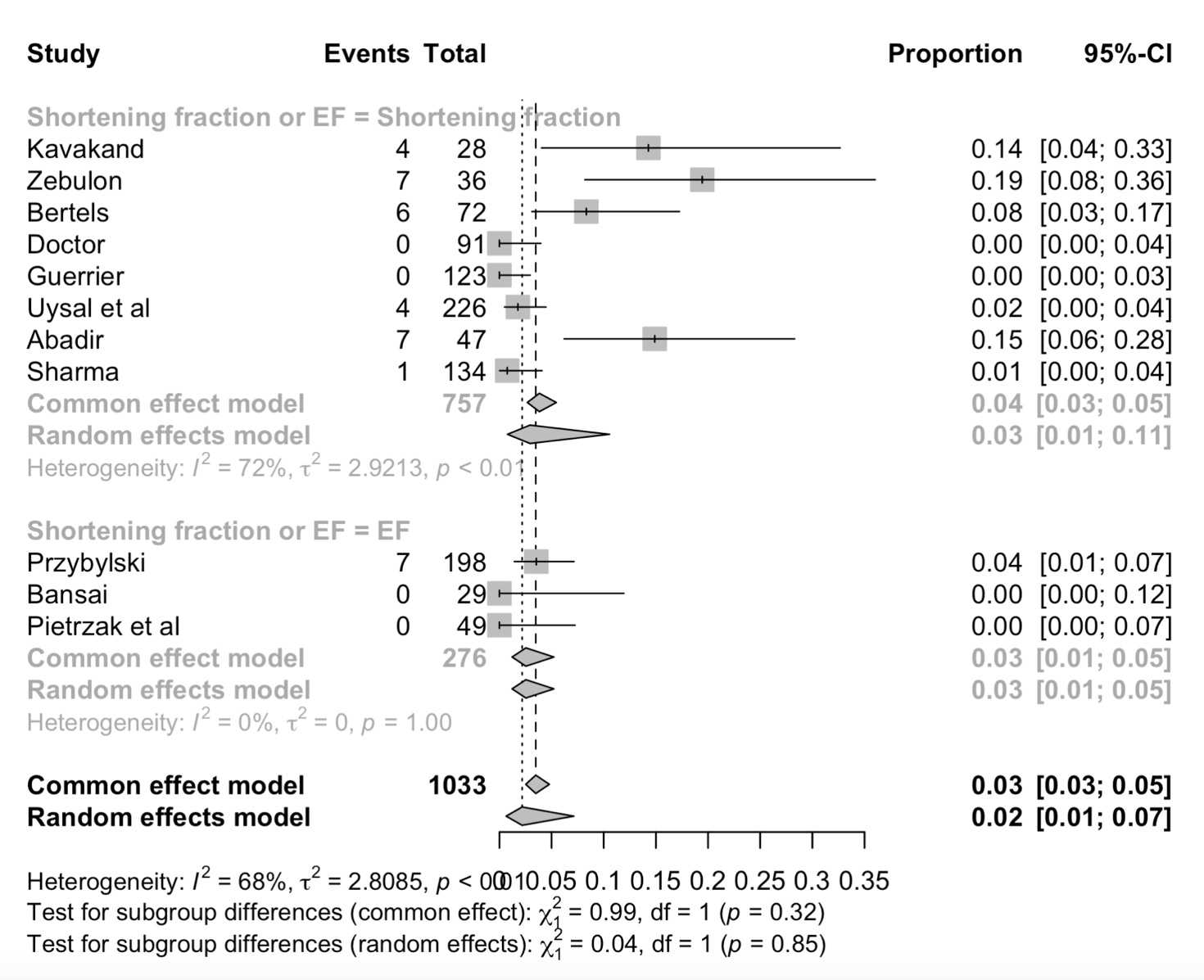


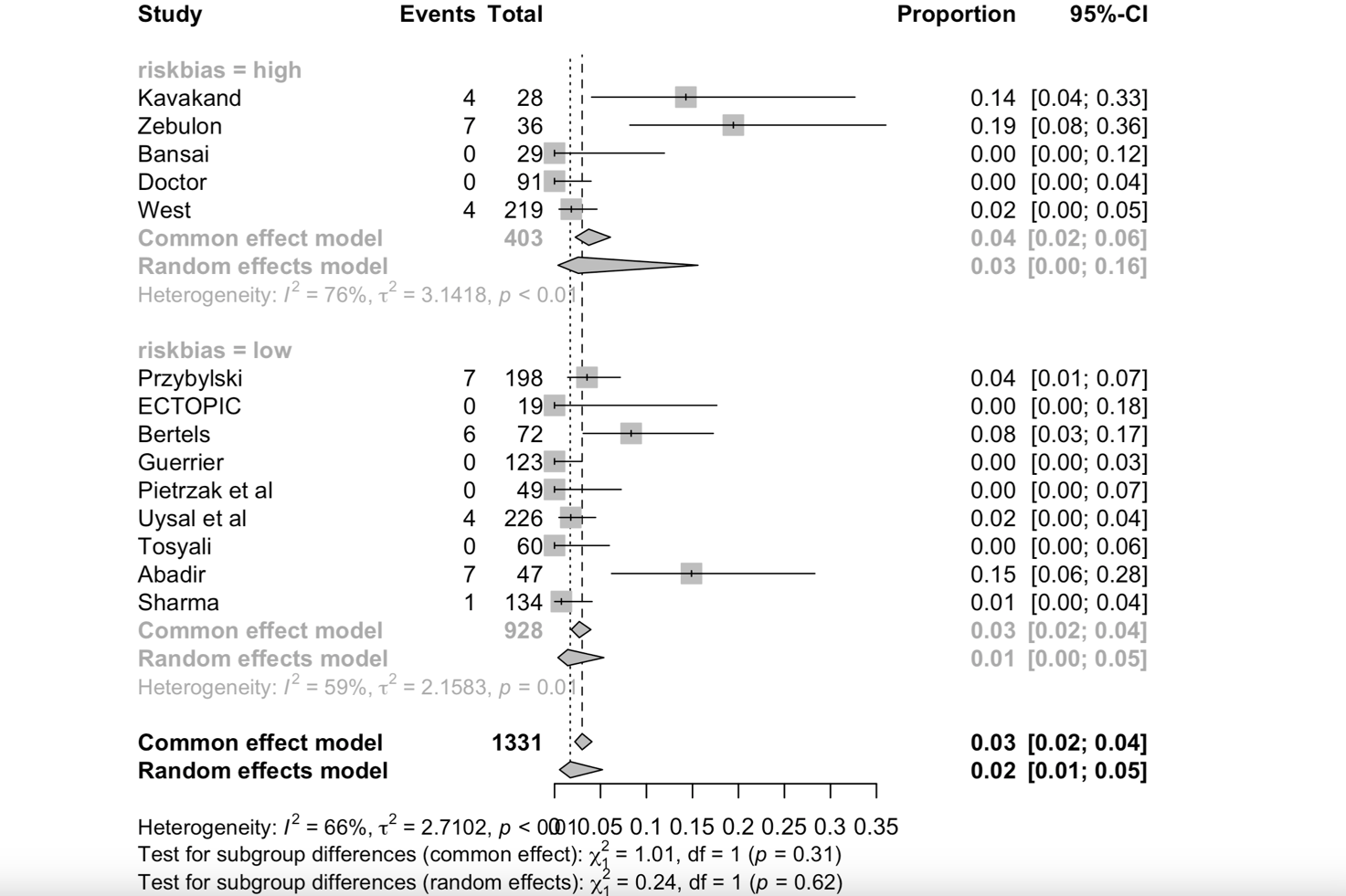


***Supplemental Figure 3:*** *Funnel plot of the effect estimates of prevalence of PVC induced CMP*


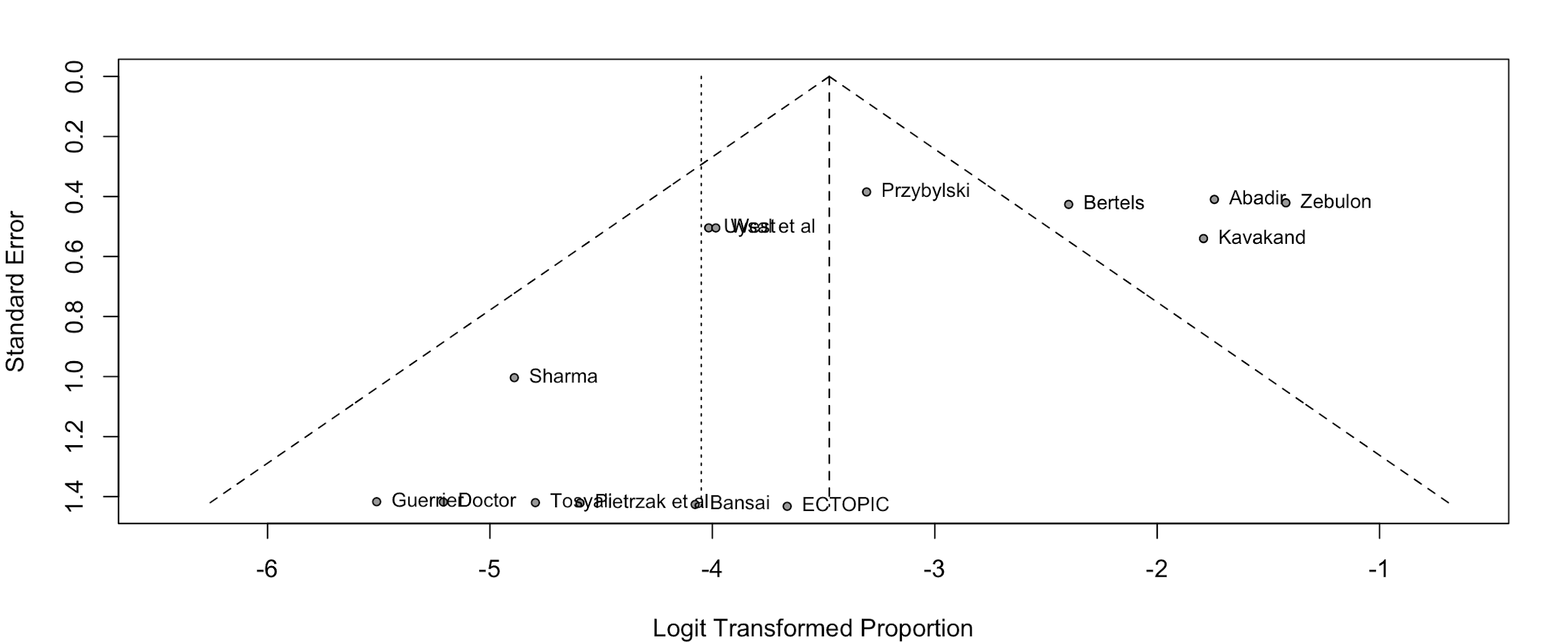


***Supplemental Figure 4:*** *Bias-corrected estimated of pooled prevalence according to Duval and Tweedie trim-and-fill method (a) complete cohort (b) after removal of the outliers evidenced in influence analysis*

(a)**
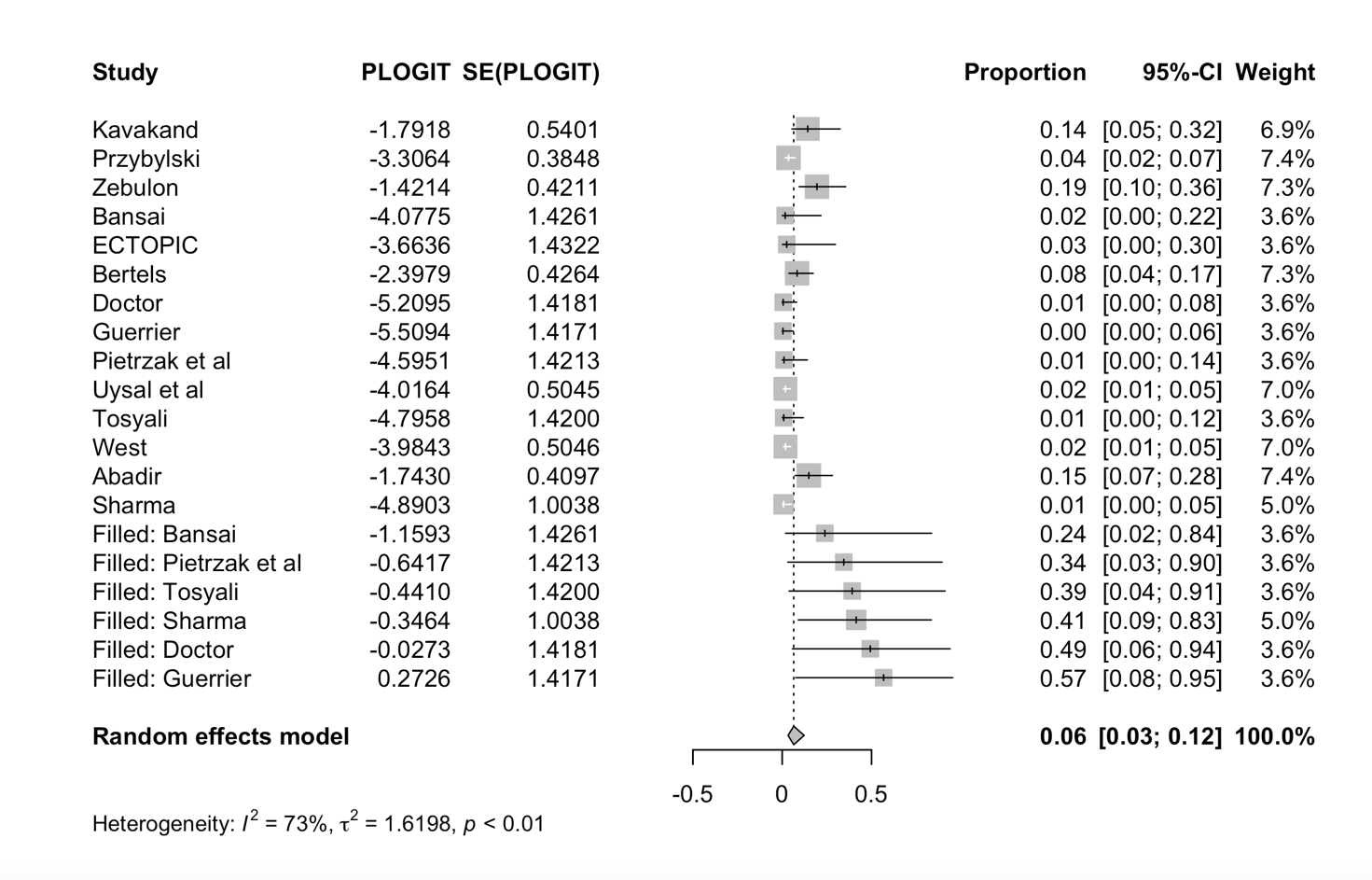
**

(b)


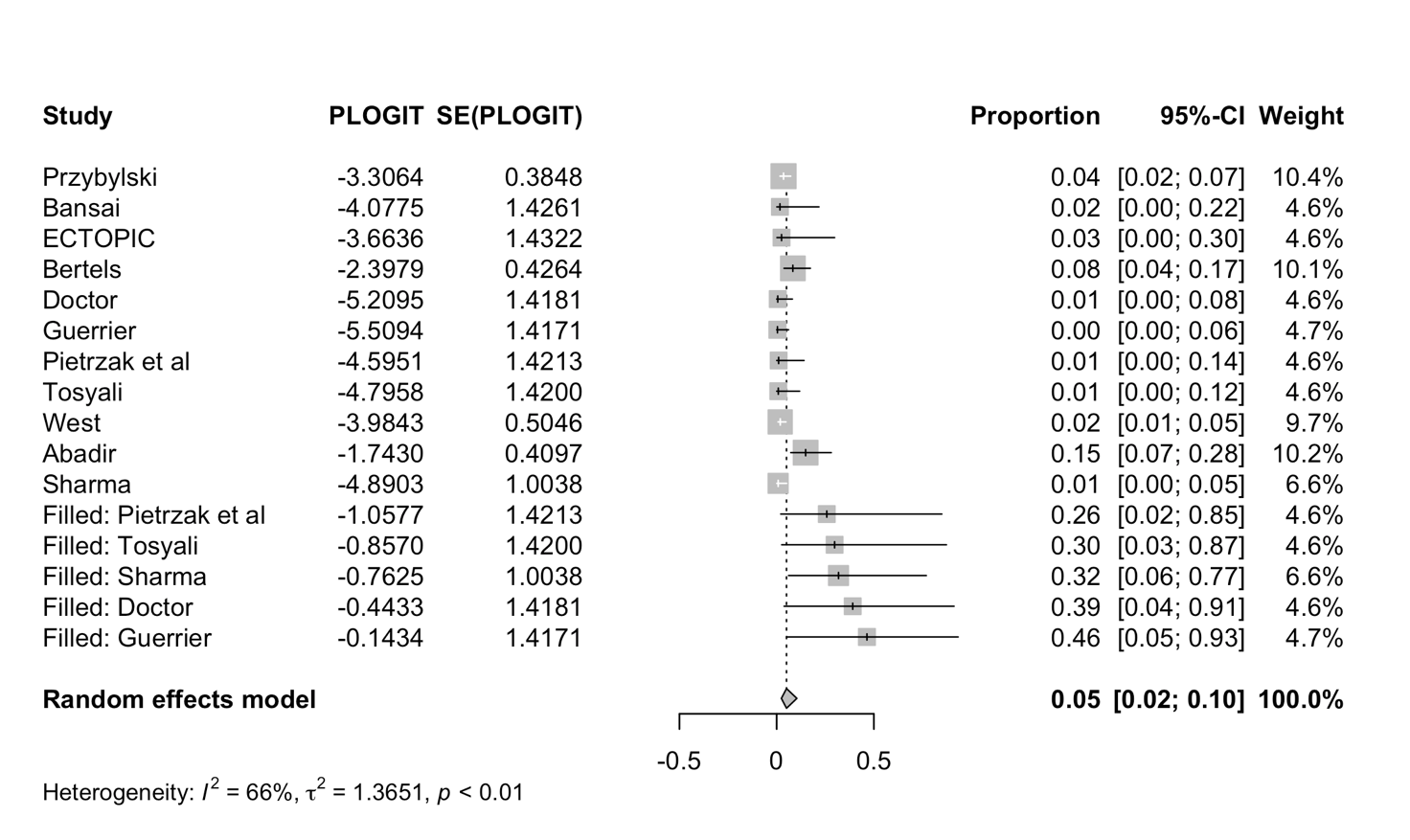


***Supplemental Figure 5:*** *Subgroup analysis of the mean burden difference*

**
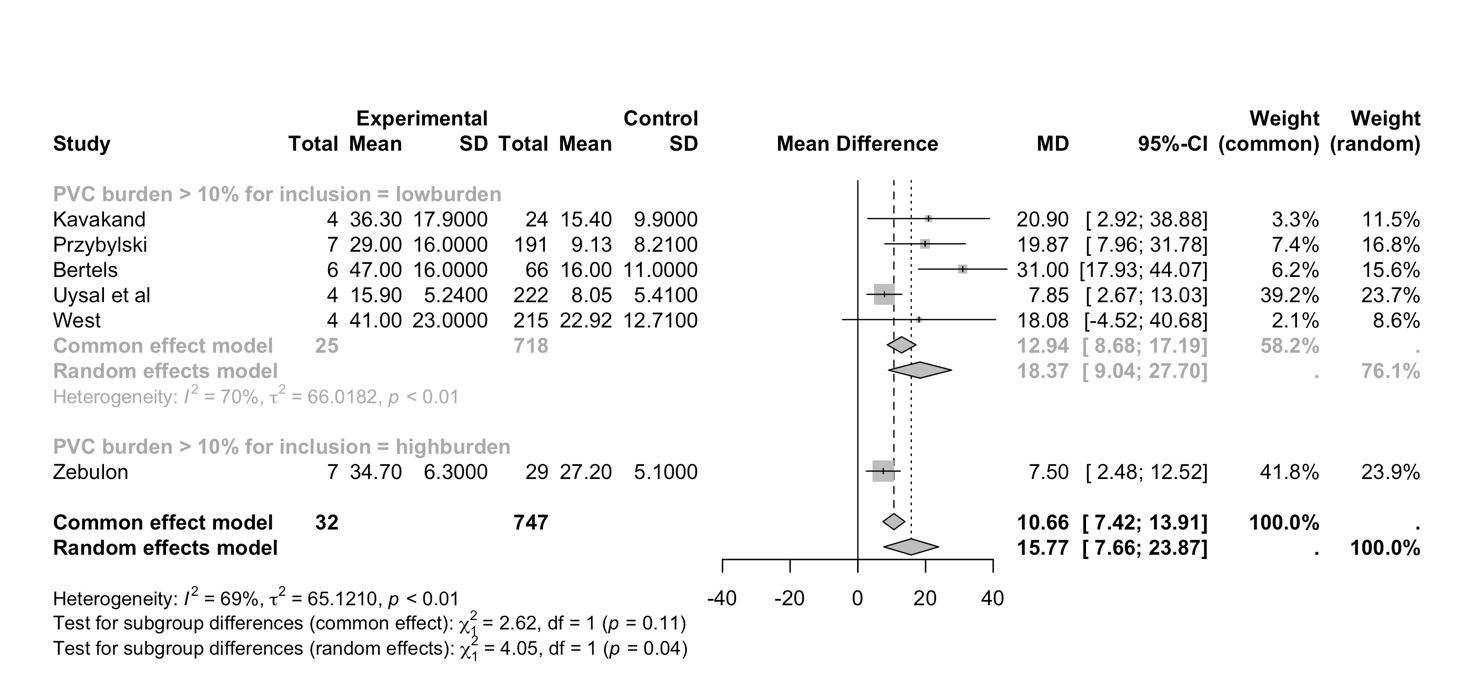
**

**
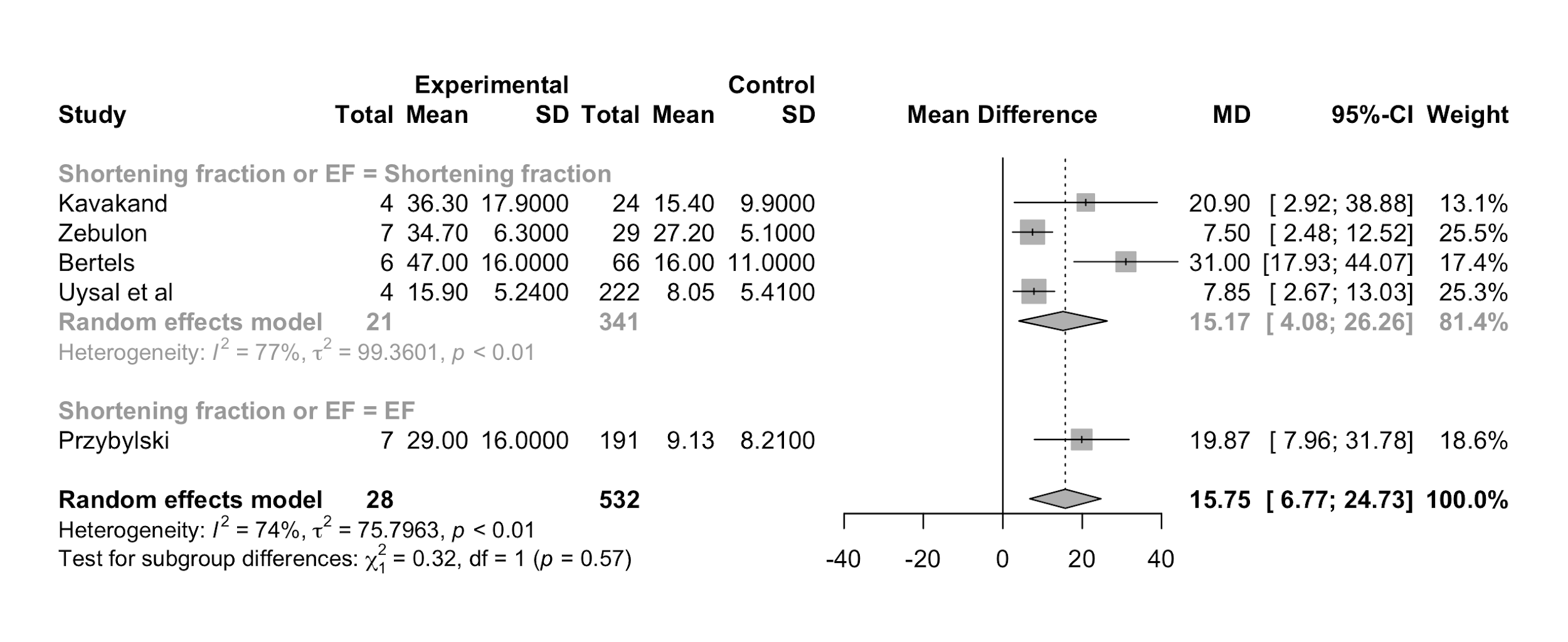
**

**
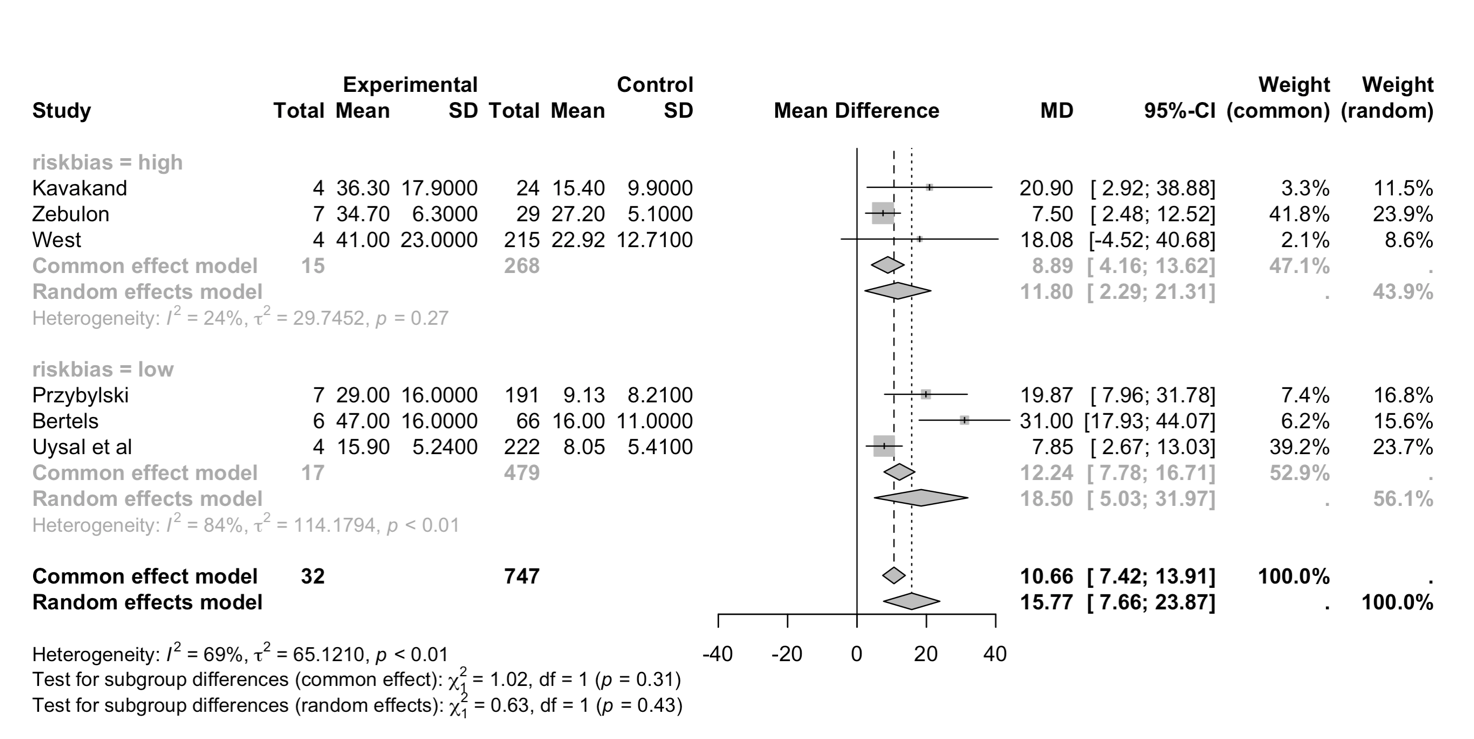
**

***Supplemental Figure 6:*** *Forest plots of the odds ratios of LBB-PVC (a) and RBB-PVC (b) on the risk of PVC-induced CMP*


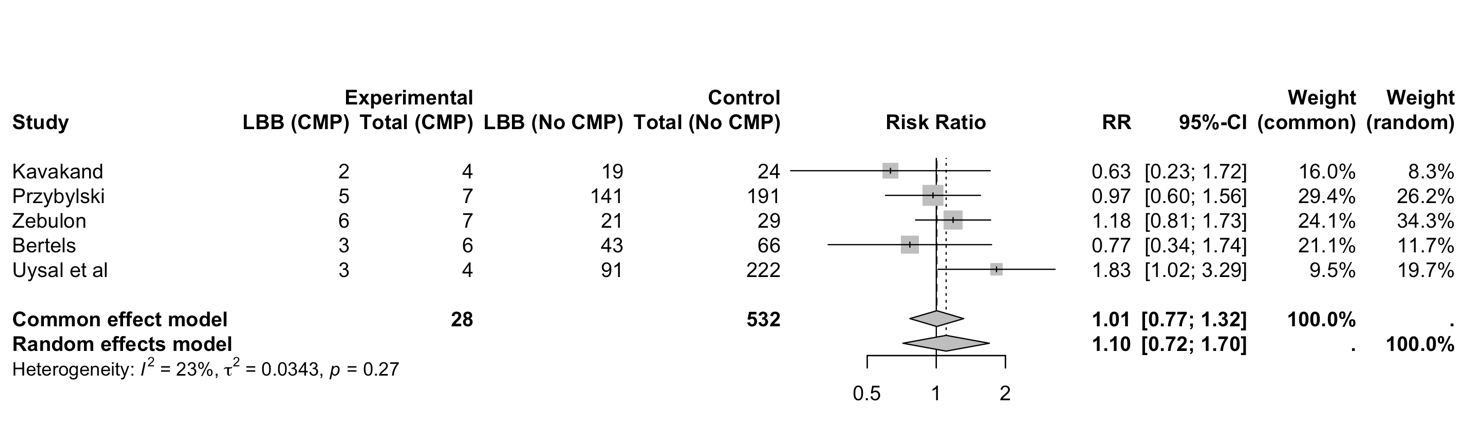


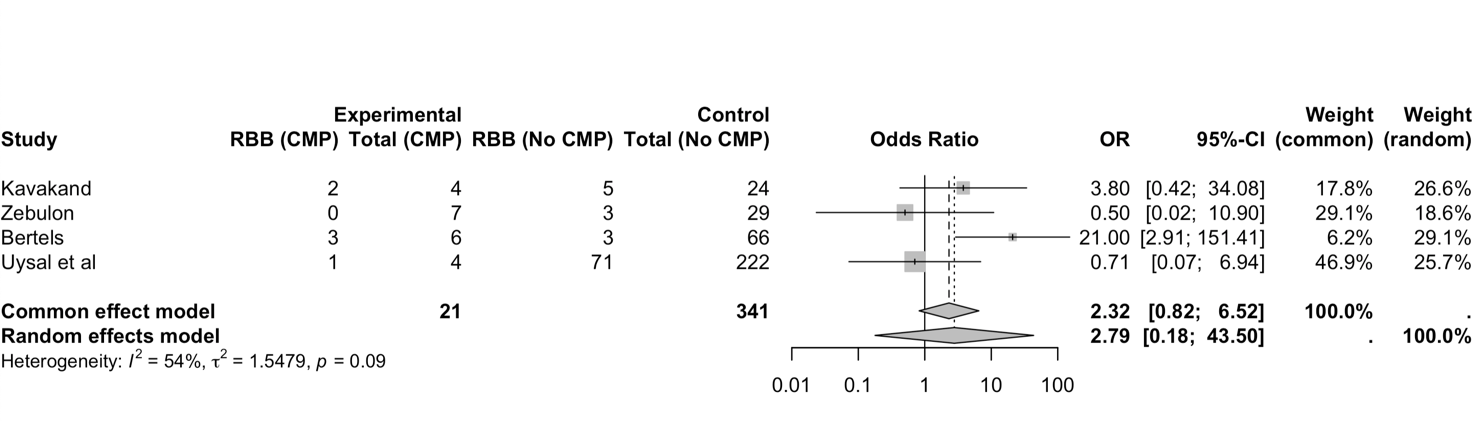


***Supplemental Figure 7:*** *Forest plot of the mean difference estimates of QRS duration according to the presence PVC induced CMP*


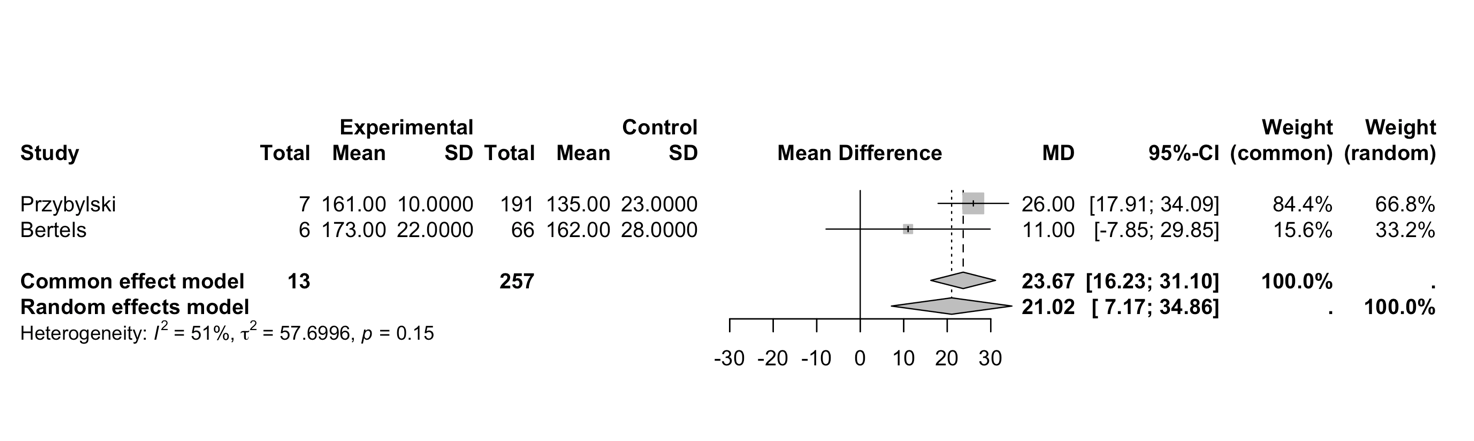

Supplement: euaf167_Supplementary_Data [file euaf167_supplementary_data.docx]
